# Forecasting the future number of pertussis cases using data from Google Trends

**DOI:** 10.1016/j.heliyon.2021.e08386

**Published:** 2021-11-12

**Authors:** Dominik Nann, Mark Walker, Leonie Frauenfeld, Tamás Ferenci, Mihály Sulyok

**Affiliations:** a Institute of Pathology and Neuropathology, Department of Pathology, Eberhard Karls University, University Clinics Tübingen, Tübingen, Germany; b Department of the Natural and Built Environment, Sheffield Hallam University, Sheffield, United Kingdom; c Physiological Controls Research Center, Óbuda University, Budapest, Hungary; d Corvinus University of Budapest, Department of Statistics, Budapest, Hungary; e Institute of Tropical Medicine, Eberhard Karls University, University Clinics Tübingen, Germany

**Keywords:** ARIMA, Forecasting, Surveillance, Pertussis, Infodemiology

## Abstract

**Background:**

Alternative methods could be used to enhance the monitoring and forecasting of re-emerging conditions such as pertussis. Here, whether data on the volume of Internet searching on pertussis could complement traditional modeling based solely on reported case numbers was assessed.

**Methods:**

SARIMA models were fitted to describe reported weekly pertussis case numbers over a four-year period in Germany. Pertussis-related Google Trends data (GTD) was added as an external regressor. Predictions were made by the models, both with and without GTD, and compared with values within the validation dataset over a one-year and for a two-weeks period.

**Results:**

Predictions of the traditional model using solely reported case numbers resulted in an RMSE (residual mean squared error) of 192.65 and 207.8, a mean absolute percentage error (MAPE) of 58.59 and 72.1, and a mean absolute error (MAE) 169.53 and 190.53 for the one-year and for the two-weeks period, respectively. The GTD expanded model achieved better forecasting accuracy (RMSE: 144.22 and 201.78), a MAPE 43.86, and 68.54 and a MAE of 124.46 and 178.96. Corrected Akaike Information Criteria also favored the GTD expanded model (1750.98 vs. 1746.73). The difference between the predictive performances was significant when using a two-sided Diebold-Mariano test (DM value: 6.86, *p* < 0.001) for the one-year period.

**Conclusion:**

Internet-based surveillance data enhanced the predictive ability of a traditionally based model and should be considered as a method to enhance future disease modeling.

## Highlights

• Pertussis-related Google Trends Data (GTD) showed a weak but significant correlation with the reported weekly number of pertussis cases.
• We fitted a SARIMA models to estimate reported weekly pertussis case numbers
• The GTD-expanded models achieved significantly better predictive accuracy than the traditional model over a one-year-period.
• Corrected Akaike Information Criteria also favored the GTD-Expanded SARIMA model.
• The use of GTD should be considered as a method to enhance pertussis forecasting.

## 1 Introduction

*General introduction to pertussis*: Pertussis, also known as whooping cough, is an acute, highly contagious respiratory tract illness. It results from infection with Gram-negative bacteria of the *Bordetella* genus; predominantly *B. pertussis*, and to a lesser extent *B. parapertussis* [1]. The consequences of infection can be severe, especially in infants. In infants, pertussis is associated with high levels of mortality, particularly in those under three months of age. The number of infected infants requiring hospitalization and intensive care remains high [2, 3].

Historically, the disease was a major cause of childhood morbidity and mortality. Pertussis is now a vaccine-preventable disease. Whole-cell vaccines that induce long-lasting protective immunity were introduced in the 1950's. These were replaced in the 1990's by acellular vaccines. The effectiveness of these declines over time and they possibly provide less protection against carriage. However, acellular vaccines are considered associated with fewer side effects [4].

Despite widespread vaccination, pertussis has made a comeback; there has been a resurgence in incidence in many developed countries in recent decades [5]. In 2018, the countries reporting the highest number of pertussis cases were China (22,507), India (13,208), Germany (12,907), followed by Australia (12,555) [6]. However, the number of reported cases represents only the tip of the iceberg; in developed countries many cases go unrecognized meaning the true extent of pertussis infection is unclear. In developing countries pertussis continues to be a major cause of infant and childhood mortality. In 2014 there were an estimated 24 million cases and 160,000 deaths from pertussis in children younger than five years old worldwide [7].

Pertussis is probably underreported, especially in adults, because of the wide variability in disease presentation [8, 9]. Initial symptoms are non-specific and mild, often mirroring those of other minor illnesses, meaning misdiagnosis easily occurs. Previously vaccinated individuals experiencing infection exhibit atypical symptoms; another important reason why pertussis is often easily missed [10].

Several factors possibly account for the resurgence in pertussis incidence in recent years, including the rise of vaccine hesitancy, the waning in the effectiveness of acellular vaccination over time, and better methods of infection detection through PCR [11]. An epidemiological shift in infection may also have occurred, with fewer children becoming infected but more adolescents, adults, and infants [12]. This may be because the effectiveness of the newer acellular vaccination declines over time, meaning that children, vaccinated at preschool age remain well protected, but adolescents and adults not recently vaccinated are more likely to experience mild infection. They may then transmit the infection to unvaccinated infants [5]. Despite high vaccination rates, periodic epidemics occur, suggesting that vaccination is effective at preventing the worst of the symptoms but not in preventing further transmission of disease.

*Background to the use of Internet search data for disease surveillance:* The re-emergence of pertussis in recent decades indicates that there is a need to enhance monitoring and forecasting using novel methods. Using data related to Internet searching is one such potential avenue for investigation. The Internet is now the primary information source for those seeking health care information; with Internet searching creating large amounts of data relating to the topics sought for [13].

Researchers were quick to investigate the use of such data in the monitoring and forecasting of infectious diseases [Reviewed: 13&ndash;16]. A notable, and well known, early attempt was Google Flu Trends (GFT), which attempted to use such data to predict likely influenza occurrence [17]. Although offering great potential, GFT was plagued with problems, primarily due to overfitting [18]. Internet search data has been used in numerous later studies for various other conditions [19, 20, 21].

Several studies have examined the potential utility of such Internet search data in the surveillance of pertussis. One of the first was a study which used Google search predictors to model pertussis in California [22]. A methodology similar to that employed by GFT was utilized, with data being selected from Google Trends with the use of Google Correlate, then Pearson correlation selecting those used in modeling. Predictors were added to a linear model in a stepwise fashion. The authors concluded that using Google Trends “cautiously offers a complementary, real-time signal to enhance pertussis surveillance”.

Other studies examining the effectiveness of Google Trends data (GTD) in the modeling of pertussis include a study forecasting pertussis incidence for mainland China using Auto Regressive Integrated Moving Average (ARIMA) and Exponential Smoothing time series modeling [23]. A later study used SARIMA modeling, the seasonal variation of ARIMA, to show that GTD could enhance models based solely on case numbers for Australia [24]. A later study by the same lead author used lagged weather variables, as well as Internet search data [25]. Predictions made using SARIMA modeling and regression trees were improved by elegantly integrating online activity data along with traditional surveillance information, leading to more accurate forecasting. Another study combined SARIMA modeling with Nonlinear Autoregressive Network (NAR) modeling, finding that combining these approaches resulted in better modeling [26].

Other studies have described the relationship between searching volume and pertussis incidence, but not performed modeling. For example, a recent study examined pertussis cases and deaths across Europe, finding a strong correlation with GTD [27]. However, no forecasting was performed. Another examined the correlation between GTD and pertussis case number across the U.S.A.; strong correlations were found, but the strength of these varied greatly between states [28].

Here, the aim was to confirm that Internet search data could enhance modeling of pertussis, but in a European context. Can the predictive accuracy of traditional models based solely on case numbers be enhanced with the addition of GTD? Comparing models both with and without GTD avoids the pitfalls seen in previous studies, which have modeled using only Internet data. Earlier attempts using solely search data were plagued with problems when fitting search activity data into seasonal patterns. It is important to emphasize that this study uses search data in addition to (and not in lieu of) the reported case numbers used in traditional forecasting.

Here, ARIMA modeling was chosen as the method with which to examine this research question; this is a widely used and well understood method of modeling [29, 30], often favored in disease forecasting. This method has also been used in other pertussis modeling studies, thus allowing some comparison [24, 25, 31]. As pertussis cases are likely underreported, the use of Internet-based resources may provide greater clarification of the underlying epidemiological trends than is apparent in reported case numbers.

## 2 Methods

*Data:* The weekly number of pertussis cases in Germany was downloaded from the database of the Robert Koch Institute (RKI), the German organization responsible for the recording of infectious diseases (SurvStat@rki 2.0; https://survstat.rki.de/Content/Query/Create.aspx) for the dates from the 13th of April 2014 to the 17th of March 2019. A query was created on the 8th of April 2019, with ‘Keuchhusten’ as the disease (the usual German term for pertussis), and ‘Jahr und Meldewoche’ (German for ‘Year and Week of notification’) as the time unit. Thus, data from a total of 258 weeks were obtained.

Data on Internet searching on pertussis was obtained from Google Trends on the 8th of April 2019 (https://trends.google.com/trends/), with ‘Keuchhusten’ as the search term, with ‘Germany’ as country, and ‘last five years’ chosen as timespan category. The values are integers of the relative weekly search ‘volume’, where 100 represents the highest search activity.

Both datasets were divided into training and validation parts. Training data covered the period from the 13th of April 2014 to the 26th of March 2018 and thus contained 207 data points. The validation dataset was from the 26th of March 2018 to the 17th of March 2019 and contained 52 data points.

*Time series decomposition:* Data collected over time can be broken down to show the underlying cyclical or seasonal patterns, trends, and random noise it contains. Such decomposition allows trends and patterns, which might otherwise be obscured, to be identified and studied. The weekly pertussis case number was decomposed to reveal trend and seasonality components using multiple 'Seasonal and Trend decomposition using LOESS' (STL) decomposition [32, 33]. LOESS (locally estimated scatterplot smoothing) creates smooths for each component of the time series.

The STL method was preferred due to its flexibility; patterns present within time series do not have to occur within standard time frames, such as monthly, with use of this method. This is the case for pertussis, where regular cycles occur, but not within standard weekly or monthly time frames. Another advantage of using STL is that decomposition is not overly affected by the presence of outliers in the data.

*Contemporaneous correlation:* The level of contemporaneous correlation between the GTD and weekly case numbers was ascertained in an exploratory investigation, being assessed with Kendall τ correlation. Normality of pertussis case number data was checked with a Shapiro-Wilk test.

*Model selection:* Time series data was modeled using a seasonal autoregressive moving average model, after differentiation if needed (SARIMA) [34], similar to the method described previously [35, 36].

The necessary steps of differentiation needed to achieve stationarity was ascertained with a KPSS test for the non-seasonal part (up to 2 differences) and with a seasonal strength test for the seasonal part (with maximally 1 differentiation). In the instance that models with and without search volumes required a different number of differentiations, the bigger number was forced in both cases, so that both models used the exact same dataset for learning.

Selection of optimal seasonal and non-seasonal autoregressive and moving average order on the stationary process was performed automatically. The optimal model was selected by minimizing the corrected Akaike Information Criterion (AICc) (order was maximally five non-seasonally and maximally two seasonally; the selection was done in a stepwise fashion) [34]. The whole procedure was then repeated, entering GTD into the original model as an external regressor, expanded with B-splines to handle non-linearity.

*Residual diagnostics:* Residual diagnostics were checked visually for autoregression, and to check the pattern of distribution. Autoregression was also assessed with the Ljung-Box test.

*Forecasting:* The predictive performances of the original and GTD expanded models were compared using RMSE (root-mean-square error), and MAPE (mean absolute percentage error). Predictions were directly compared using the modified Diebold-Mariano test [37].

*Validation:* We also performed a second, short term forecast for the first two weeks of the validation dataset. Additionally, evaluation on a rolling forecasting origin was performed [33], using the whole dataset, a forecasting horizon of one, and a window width of 100 weeks.

Prediction errors of the two models (with and without GTD) were compared visually and using RMSE values.

All statistical analyses were performed in R version 3.4.4 using the forecast package version 8.7 [33]. The script and the dataset are available online at https://github.com/msulyok/GoogleTrendsPertussis, and detailed in the Supplementary Material.

## 3 Results

**Figure 1:**
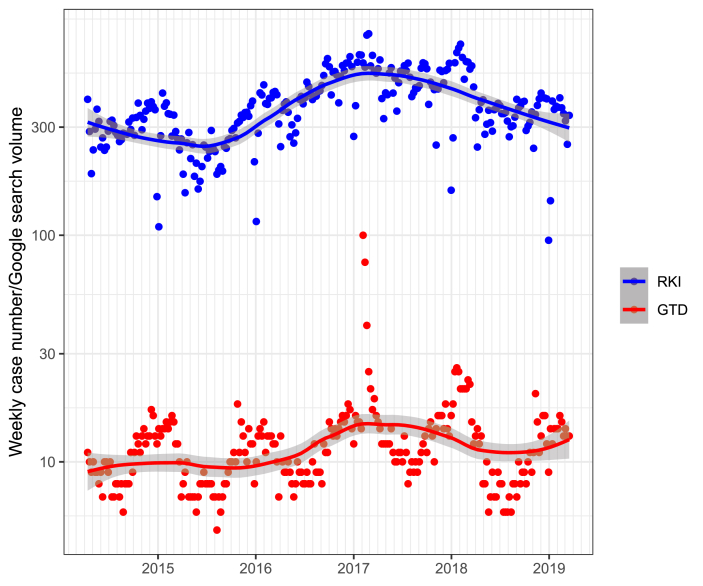
Weekly pertussis case numbers and Google Trends data; weekly pertussis case numbers are shown with a blue curve; relative Google Trends search volumes are shown with a red curve. LOESS smoothers representing trend lines with 95% CI-s (shown with the same colour).

*Descriptive statistics:*Figure 1 shows the weekly reported number of pertussis cases over the time period examined and the corresponding patterns in Internet searching on ‘Keuchhusten’ from Google Trends. Clear seasonal patterns in pertussis case numbers are apparent through observation; pertussis case numbers rise in Winter months and decline in Spring. An overall rise in cases is apparent over the time period examined. GTD showing volumes of Internet searching on pertussis shows a similar seasonal pattern with clear increases during Winter months.

**Figure 2:**
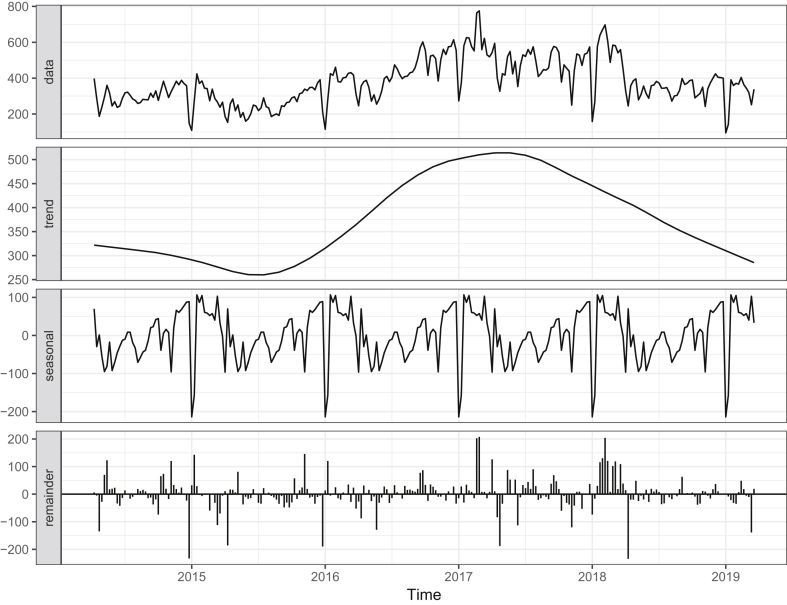
STL time-series decomposition of weekly pertussis case numbers; trend, seasonal and random components of the reported case numbers are shown.

*Time series decomposition:* After STL decomposition of weekly case numbers, these patterns in the overall trend and seasonality were even more apparent (Figure 2). Decomposition revealed an increasing trend throughout 2016 and into 2017, with a decrease in 2018. The overall trend peaked in 2017. The peak of the seasonal component corresponded to the month of February. Annually, case numbers rose during the Winter months.

*Contemporaneous correlation:* The Google search volume showed a significant contemporaneous correlation with reported weekly case numbers (τ = 0.398; p < 0.001) (Figure 1).

*Model selection:* Examination of the pertussis time series clearly shows that it is non-stationary, and thus that differentiation is required. The results of KPSS testing indicated that taking the first differences was required to achieve stationarity. Thus 1 level of differentiation was used for both non-seasonal and seasonal data.

**Figure 3:**
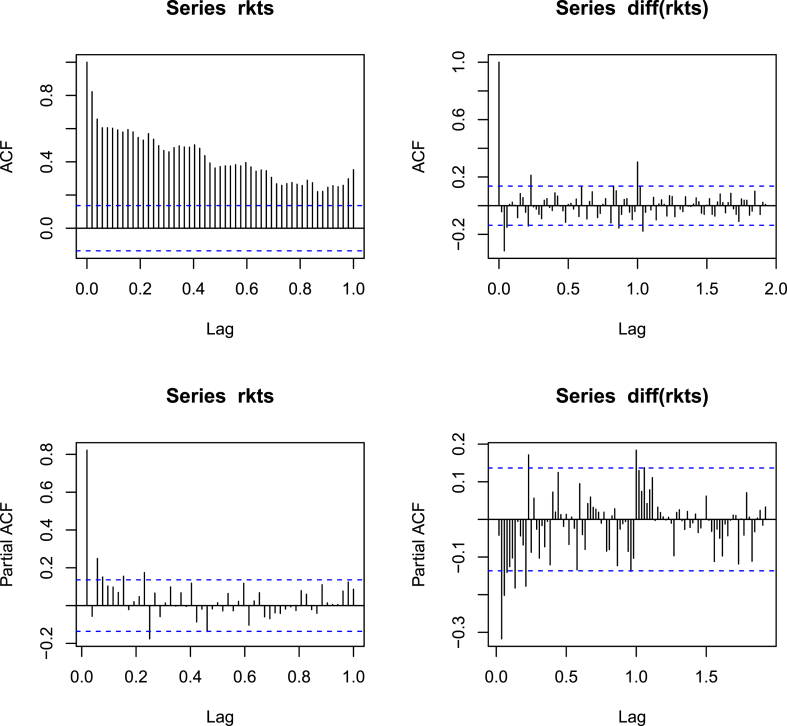
Autocorrelation (ACF) and partial autocorrelation function (PACF) plots of the reported weekly pertussis case numbers. Upper left: Undifferentiated ACF; Upper right: Undifferentiated PACF; Bottom left: Differentiated ACF; Bottom right: Differentiated PACF.

Examination of the ACF plot for the pertussis time series confirmed that pertussis incidence is clearly seasonal in nature, as indicated by the strongly significative peak at lag 1.00. This suggests a strong seasonal component occurring on a 12-monthly basis. Additionally, the presence of geometric decay observable in both the ACF and PACF plots suggests that a seasonal moving average component is required when establishing an ARIMA model (Figure 3).

This was confirmed by automatic selection of the optimal model; which identified a SARIMA (1,1,3) (0,1,1)[52] as being optimal.

The detailed model selection process is shown in the supplementary material. The optimal model had a non-seasonal component with single autoregressive, three moving average, and one differentiation order. The seasonal component had zero autoregressive, a single moving average, and single differentiation order (i.e., it was SARIMA (1,1,3) (0,1,1)[52]). The number of autocorrelation and moving average orders selected was also confirmed visually using the ACF and PACF plots. These indicate modeling requiring both AR and MA components. Significant coefficients for the traditional model were the autoregressive term, the first and third moving average term, and the seasonal moving average term. When the spline described external regressor of GTD was added, autoregressive order 1 and 2, moving average order 1 and 2, and the seasonal moving average term were significant.

The AICc of the traditional model was 1750.98, while that of the GTD-extended model was more favorable: 1746.73 (Table 1).

**Table 1:** Summary of the model using case numbers only, compared with the GTD-enhanced model.

	Model without GTD	Model with GTD
Model formula	ARIMA (1,1,3) (0,1,1)[52]	ARIMA (1,1,3) (0,1,1)[52] with errors
AICc	1750.98	1746.73
Training set RMSE	57.55	54.86
Validation set RMSE (for 52 weeks; for 2 weeks)	192.65; 207.8	144.22, 201.78
Validation set MAPE (for 52 weeks; for 2 weeks)	58.59, 72.1	43.86, 68.54
Validation set MAE (for 52 weeks; for 2 weeks)	169.53, 190.53	124.46, 178.96

Covariates	Coefficient (standard error)	p-value	Coefficient (standard error)	p-value
Autoregressive order 1	0.949 (0.049)	<0.001	0.611 (0.151)	<0.001
Moving average order 1	-1.449 (0.085)	<0.001	-1.179 (0.142)	<0.001
Moving average order 2	0.095 (0.136)	0.487	-0.051 (0.149)	(0.734)
Moving average order 3	0.378 (0.075)	<0.001	0.350 (0.077)	<0.001
Seasonal moving average	-0.332 (0.121)	0.006	-0.321 ​(0.12)	0.007
Spline GTD coefficient 1			295.81 (546.06)	0.588
Spline GTD coefficient 2			208.82 (477.725)	0.662
Spline GTD coefficient 3			609.176 (493.187)	0.217
Spline GTD coefficient 4			243.98 (484.016)	0.614
Spline GTD coefficient 5			259.709 (481.836)	0.589

*Diagnostic checking:* The residuals of both models (with and without GTD) showed random walk and were normally distributed. However, significant autocorrelation was detected at lags 11 and 50 with the traditional model, and at lags 11, 42 and 44 of the GTD-extended model; the results of Ljung-Box tests for the traditional model (p = 0.28) indicated independence, but for the GTD-extended model (p = 0.048) it was significant. This suggests a certain level of autocorrelation in the GTD-enhanced model; however, this was anticipated given that the same model components were selected for with the GTD-enhanced model as with the traditional model (instead of selecting the best model based on AICc). Using the same model components throughout provided the best comparison of GTD and non GTD-enhanced models. Residual diagnostic plots are shown in the supplementary material.

**Figure 4:**
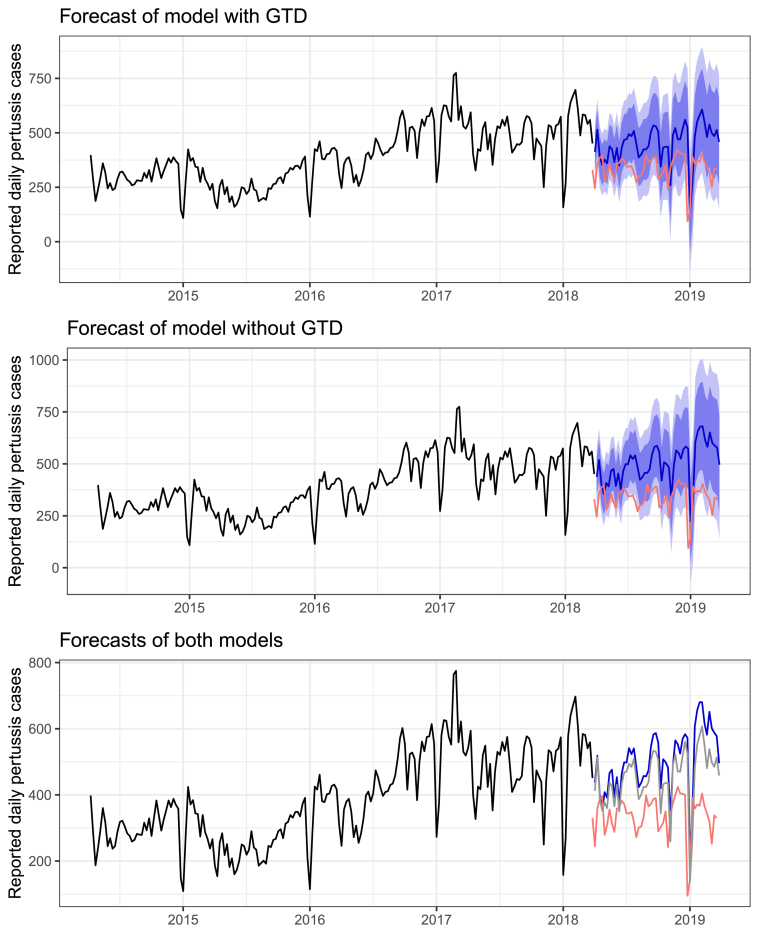
Forecasting of the optimal SARIMA model with traditional data (top); the Google Trends Data extended SARIMA (middle), and both models (bottom) compared to the reported weekly Pertussis case numbers (black curve). Shaded areas illustrate +/- 80 and 95% prediction error bounds.

*Forecasting:* Forecasted values for the one-year period for both models are presented graphically, together with the weekly pertussis case numbers in Figure 4. Predictions of the traditional model, comparing forecasted values with the reported ones, resulted in a residual mean squared error (RMSE) of 192.65, and mean absolute percentage error (MAPE) of 58.59. The values from the model expanded with Google search data achieved a better forecasting accuracy (RMSE: 144.22, MAPE: 43.86). The two-sided Diebold-Mariano test showed significant differences between the two predictive performances (DM value: 6.86, *p*-value < 0.001).

For the shorter, two-weeks interval we obtained similar results. The RMSE, MAPE and MAE values were more favorable with the GTD-extended model (Table 1), however the predictive performance was not significantly different (DM value: 1.4, *p*-value 0.39).

**Figure 5:**
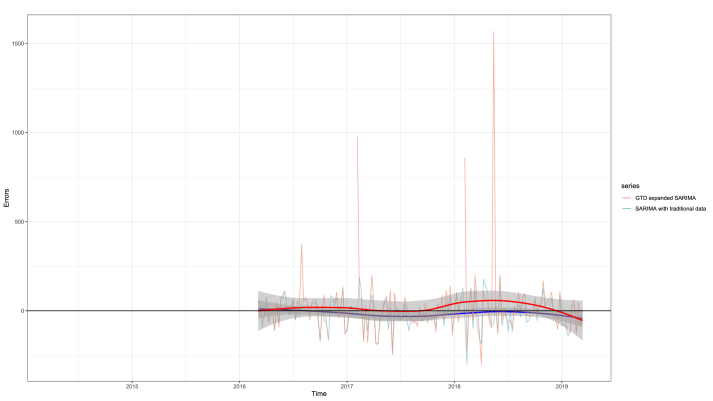
Evaluation on a rolling forecast origin-comparison of the SARIMA models. Note the peak in errors caused by an outlier at the beginning of 2017, and favorable effect of smoothing (less distance from the horizontal 0 error line).

*Validation:* Evaluation on a rolling forecast origin revealed an RMSE of 82.8 for the traditional model and 30490 for the GTD-extended one. The smoothed error terms showed however similarity to the traditional one (Figure 5)

## 4 Discussion

*Study findings:* Here, patterns in pertussis incidence and corresponding levels of Internet interest from Google Trends were examined for Germany over a four-year time period. A significant contemporaneous correlation between GTD and the reported weekly case numbers of pertussis was observed. Decomposition revealed the cyclical character of pertussis. The seasonal nature of pertussis occurrence was apparent, with a notable peak in reported cases in the Winter months. An increasing overall trend was also apparent. Reported cases of pertussis peaked in the Winter of 2017 in Germany; a corresponding increase in the trend can be seen up to this period. This contrasts with other studies, which report peaks in late summer and autumn [38, 39].

The predictive accuracy of a forecasting model for pertussis was enhanced by the inclusion of data relating to the volume of Internet-based searches about the disease. Examination of the pertussis time series indicated that a SARIMA (1,1,3) (0,1,1)[52] model was the most accurate. Comparison of AICc indicates that models using either pertussis case numbers only, or with the addition of GTD have similar qualities. However, the predictive accuracy of the GTD-enhanced model was greater, as seen by the improvement in RMSE, MAPE and MAE values. Overall, the GTD-extended model proved to be superior not just in terms of model quality measures, but in the significant difference in predictive performance also, as indicated by the modified Diebold-Mariano test for the one-year period.

*German context:* In line with other European countries, incidence rates for pertussis have risen in recent decades in Germany. According to WHO, 12,907 cases were reported in 2018, the third highest country case number in the world [6]. Could this be a legacy of previous poor rates of vaccination? Before unification in 1989, rates of vaccination against pertussis varied notably between East and West Germany [40]. In East Germany vaccination rates were high and reported case number low (<1 case/100,000/year). Whereas in West Germany concern about the safety of whole-cell vaccination [41] resulted in lower vaccination rates, and thus a correspondingly higher number of estimated cases (160–180/100,000/year). Today, vaccination against pertussis in Germany is recommended by the Standing Vaccination Committee. Vaccination rates are comparable to those seen in other European countries; 94,9% for children beginning school in 2015 [42], but only 55% for the booster given at between nine and 17 years of age 42 [43].

*Wider context:* Pertussis remains of considerable epidemiological and economic concern worldwide. Pertussis in infants is associated with great morbidity and mortality, frequently requiring hospitalization and intensive care [2, 3, 44]. In many developed countries there has been an increase in pertussis incidence in recent decades [45]. The epidemiological profile of those affected has altered; with infection becoming more common in adolescents and adults [47]. Those experiencing infection have often been previously vaccinated, and experience milder often atypical symptoms [10]. It has been suggested that this epidemiological shift has occurred due to increased vaccination of infants, *Bordetella* strains that produce greater levels of pertussis toxin becoming more common, or due to the introduction of less long-lasting acellular vaccination in the 1990's. Pertussis is probably underreported in Germany and elsewhere [8, 9, 46, 47], further illustrating the importance of, and the requirement for, precise data on this condition.

*SARIMA modeling:* Here, seasonal ARIMA modeling was purposely chosen as the vehicle to ascertain whether the inclusion of Internet based search data could be used to enhance modeling using solely pertussis case numbers, as is traditionally the case. Since its widespread adoption from the 1970's onwards, ARIMA modeling, which combines autoregressive, integrative, and moving average components, has become one of the most reliable methods of time series analysis for infectious diseases [48]. The seasonal version of the ARIMA model is ideal for a condition such as pertussis which, as was shown here, is seasonal in nature. SARIMA modeling provides better forecasting accuracy when the time-series being studied is non-stationary in nature, and exhibits clearly seasonal trends [34].

Thus, although more advanced modeling exists, such as Neural Networks, SARIMA provides the obvious choice to answer our research question [24, 25, 26].

Additionally, it permits comparison with other studies using SARIMA. Our findings extend the excellent work of Zhang et al. [24]. Initially testing various search terms, they finally selected “whooping cough” as it showed the highest correlation with the incidence numbers. They then used data from Google Trends relating to this search term to extend their SARIMA (2,0,2) (1,0,0) model. The external regressor improved BIC and RMSE and showed an overall agreement of 81% (sensitivity: 77% and specificity: 83%) to the validation dataset (monthly incidence for 36 months). However, they did not report the predictive performance of the model without GTD, and thus provided no comparison with the GTD-extended model. Since the more favorable BIC and better fitting to the training dataset does not necessarily mean a better fit to the validation dataset, in our opinion, a direct comparison would be warranted. A major strength of our study was that such a direct comparison of forecasting accuracy was conducted. Another advantage is that here data was examined at a higher resolution (weekly compared to monthly), meaning there were more observations to train and validate the models. Zhang et al. [24] used data over a longer time span (108 months training; 36 months validation) than here (47 months training; 12 months validation); but data was examined only monthly.

*Other types of modeling:* Already mentioned is a study which utilized linear regression modeling, fitting data from Google Trends in a methodology similar to GFT [22]. However, we believe that the method they chose; fitting linear regression models to non-linear and non-stationary time series is not as effective as using time series modelling approaches, such as ARIMA, as we have done here. The use of linear regression does not allow modeling of dynamic behavior (such as seasonality).

Another recently published work also identified online search activity as a significant correlate to pertussis incidence in Jinan city, China [25]. This modeling also incorporated meteorological data and used a SARIMA (1,0,2) (1,0,0) model and a regression-based tree model to identify cutoffs. However, a direct comparison of predictive performances was also not published.

Comparison of the SARIMA models used in previous studies [24, 25] along with the results presented here suggest that Internet search data enhances model performance. Additional modification of SARIMA models used in previous studies [24, 25] with the German pertussis data done out of personal interest, also suggests that such Internet based data enhances models, regardless of the exact structure of model chosen (Suppl. Material). That models using such data outperform ones based solely on case numbers, irrespective of geographical location (or type of search engine for the online-activity based data) is quite remarkable, and suggests the widespread utility of such data.

The nature of the GTD used here seemed to complement the SARIMA modeling for pertussis. The GTD exhibited seasonal trends, possibly enhancing the ability of the SARIMA model to anticipate trends that were likely to occur and thus make more accurate forecasts.

SARIMA models integrate seasonal components, essentially backshifting the seasonal parts of the time series [34]. This was the case here, with the pertussis dataset exhibiting clear seasonal trends. Clear trends in Internet searching are apparent from the GTD, and these appear to complement the seasonal nature of the pertussis time series, thus enhancing forecasting when integrated with it. Use of SARIMA complements other studies examining both pertussis [25] and other infectious diseases, and further confirms that seasonal modeling techniques are appropriate here.

Predictions of AR-based models can lag behind what actually occurs; they have only past observations with which to work with and thus unable to anticipate shifts in trend that may occur. Thus the advantage of SARIMA models; through integrating seasonal patterns, expected changes can be anticipated which generally results in a greater forecasting accuracy.

*Strengths and limitations:* The main strength of this study is that it quantifies and statistically compares the predictive performances of a traditional model using solely case numbers with a GTD-enhanced model. The results are also validated with different validation approaches. The emphasis is less on descriptive correlations, more on shedding light on forecasting accuracies. This contrasts with other studies which have concentrated on comparison of model types [25, 26].

The main limitation is a low number of time series observations, and that it uses only a single dataset from a single country. Validation confirming the utility of GTD in pertussis modeling could be performed using data on pertussis incidence from other countries, over different time frames, and would be a productive area of further investigation.

Of note, evaluation on a rolling forecast origin revealed a worse predictive performance for the GTD-extended model. This is clearly the result of some outliers in the GTD data. This may shed light on the main problem of such studies; the effect of outliers in online activity-based data. If the error terms were smoothed, the two curves showed a high degree of similarity. An important limitation of this internal validation method was the wide rolling window applied; this has limited the strength of this method considerably.

Another limitation is that although forecasting can predict the likely future number of notified cases of pertussis, this is nevertheless likely to be an underestimate as this condition is thought to be underreported. Thus, further, long-term validation is warranted. Additionally, GTD is probably influenced by related media activity; we suspect the peak value of 100 in 2017 is probably a result of such an effect. However, such media interest probably reflects ongoing disease activity, although with some background 'noise'. Further improvements could be made by adding more external regressors and even performing geospatial analyses. Alternative forecasting approaches like State-space models or neural networks could also be utilized.

We have to emphasize that our intention was not to establish a model to reach the highest possible predictive accuracy. The focus of this paper was rather to assess the possibility of online-activity -based data to improve traditional data-based modelling.

*Advantages of Internet based search data for disease surveillance:* This study illustrates the potential use data relating to Internet search data could play in modeling of conditions whose epidemiological characteristics mean forecasting is particularly tricky. The true level of pertussis incidence, for example, is difficult to ascertain due to the large number of asymptomatic, subclinical, or misdiagnosed cases [3]. The integration of Internet data in the modeling of such conditions could be particularly useful in mitigating such gaps in knowledge.

Several recent reviews have highlighted the growing body of research examining the use of Internet search results for epidemiological surveillance and other healthcare problems [13, 14, 15, 16]. There are many advantages of using such Internet based data. Such data is often of minimal cost or even freely available [16]. Internet search results are easy to obtain, often through simple Internet searching [14]. Such data is often up to date, with data being automatically updated thus providing real time relevance [49, 50]. The ARIMA based modeling used here is straightforward, and it is not inconceivable that desktop bound epidemiologists with minimal modeling expertise, could utilize it at a local level for small scale predictive purposes [48].

*Future research:* The aim in this study was to examine whether integration of data relating to online search activity could enhance modeling forecast accuracy. Further research could determine which modeling techniques result in the highest forecasting accuracy, and the influence such online activity data has on different model types. A good next step would be to examine the potential of the technique outlined here in other contexts. An obvious initial starting point, considering the study examined pertussis in Germany, would be to utilize such modelling at the state level. Germany is organized federally, with individual states having responsibility for epidemiology and disease control. An advantage of ARIMA based modeling is that it is simple enough to be widely understood, and thus locally based epidemiologists could integrate GTD into modeling on a regional level; state wise comparison of model predictive accuracy should be possible. Also of interest would be a comparison of model performance at both short and long term forecasting, and which models are most suitable over different time-spans.

## 5 Conclusion

In conclusion, integrating Internet search data could be a potentially promising avenue of investigation when refining traditionally based forecasting models, especially for conditions such as pertussis where the true levels of incidence are unclear. Here, the predictive accuracy of a model integrating such Internet search data, obtained from Google Trends, was contrasted with one based solely on reported cases numbers. The integration of such data into a SARIMA model was found to improve forecasting accuracy and demonstrates the potential such data sources offer for further modeling. Such data is up to date, easily accessible, and of minimal cost. The use of Internet search data could anticipate possible future rises in incidence, meaning more timely allocation of healthcare resources could occur.

## Declarations

### Author contribution statement

Dominik Nann, Leonie Frauenfeld: Conceived and designed the experiments; Performed the experiments; Wrote the paper.

Mark Walker: Conceived and designed the experiments; Performed the experiments; Analyzed and interpreted the data; Wrote the paper.

Tamás Ferenci: Analyzed and interpreted the data; Contributed reagents, materials, analysis tools or data; Wrote the paper.

Mihály Sulyok: Conceived and designed the experiments; Performed the experiments; Analyzed and interpreted the data; Contributed reagents, materials, analysis tools or data; Wrote the paper.

### Funding statement

This research did not receive any specific grant from funding agencies in the public, commercial, or not-for-profit sectors.

### Data availability statement

Data associated with this study has been deposited at https://github.com/msulyok/GoogleTrendsPertussis.

### Declaration of interests statement

Mihály Sulyok received a research grant UKT-AKF Nr. 346-0-0 unrelated to the current project. We have no other conflicts of interest to declare.

### Additional information

No additional information is available for this paper.

## Appendix A Supplementary data

pertussisfinal3112shortterm pertussisfinal3112shortterm
