# Supplementary material for: Forecasting the future number of pertussis cases using data from Google Trends
Source: Heliyon. 2021 Nov 12;7(11):e08386. doi: 10.1016/j.heliyon.2021.e08386 (PMC8605298; doi:10.1016/j.heliyon.2021.e08386)
Supplement: pertussisfinal3112shortterm [file mmc1.docx]

Pertussis Forecasting with splines and manually set differentation - REVISED-SAME MODELLS

Sulyok et al.

2020 dec 30

**library**(readr)
**library**( lattice )
**library**(splines)
**library**(forecast)

## Registered S3 method overwritten by 'quantmod':
## method from
## as.zoo.data.frame zoo

**library**(ggplot2)


masterall <- **read_delim**("pertussismeaslesmaster.csv", ";", escape_double = FALSE, col_types = **cols**(time = **col_date**(format = "%Y.%m.%d")), trim_ws = TRUE)


*#masterall[is.na(masterall$rkip)] <- 0*
**summary**(masterall)

## time pertugt impgt mgt
## Min. :2014-04-13 Min. : 5.0 Min. : 7.00 Min. : 1.000
## 1st Qu.:2015-07-06 1st Qu.: 9.0 1st Qu.:15.00 1st Qu.: 2.000
## Median :2016-09-28 Median : 11.0 Median :16.00 Median : 2.000
## Mean :2016-09-28 Mean : 12.3 Mean :16.37 Mean : 3.341
## 3rd Qu.:2017-12-22 3rd Qu.: 14.0 3rd Qu.:18.00 3rd Qu.: 3.000
## Max. :2019-03-17 Max. :100.0 Max. :42.00 Max. :100.000
## rkip
## Min. : 95.0
## 1st Qu.:284.8
## Median :365.0
## Mean :378.0
## 3rd Qu.:450.2
## Max. :775.0

**cor.test**(masterall**$**rkip, masterall**$**pertugt, method="kendall")

##
## Kendall's rank correlation tau
##
## data: masterall$rkip and masterall$pertugt
## z = 9.1644, p-value < 2.2e-16
## alternative hypothesis: true tau is not equal to 0
## sample estimates:
## tau
## 0.3980472

**library**(forecast)
gts <- **ts**( masterall**$**pertugt, start=**c**(2014, 15), end=**c**(2019, 12), frequency=52)


rkts <- **ts**( masterall**$**rkip , start=**c**(2014, 15), end=**c**(2019, 12), frequency=52)
**autoplot**(**stl**(rkts, t.window=52, s.window="periodic", robust=TRUE), range.bars=FALSE) **+** **theme_bw**() **+ggtitle**(NULL)


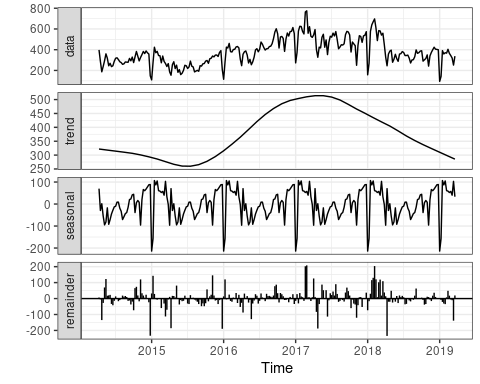


**plot**(**stl**(gts, s.window="periodic"))


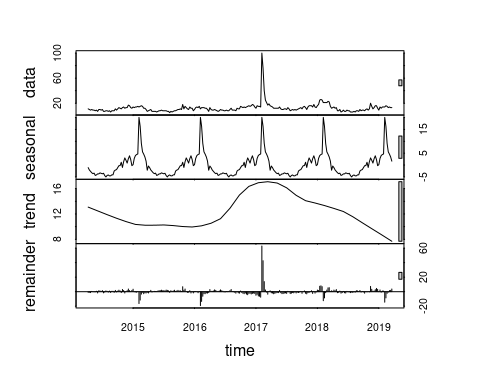


**summary**(gts)

## Min. 1st Qu. Median Mean 3rd Qu. Max.
## 5.0 9.0 11.0 12.3 14.0 100.0

**summary**(rkts)

## Min. 1st Qu. Median Mean 3rd Qu. Max.
## 95.0 284.8 365.0 378.0 450.2 775.0

gts17<-**ts**(gts[208**:**258], start=**c**(2018, 13), end=**c**(2019, 12), frequency=52)
gts<-**ts**(gts[1**:**207], start=**c**(2014, 15), end=**c**(2018, 13), frequency=52)

rkts17<-**ts**(rkts[208**:**258], start=**c**(2018, 13), end=**c**(2019, 12), frequency=52)
rkts<-**ts**(rkts[1**:**207], start=**c**(2014, 15), end=**c**(2018, 13), frequency=52)

**summary**(rkts)

## Min. 1st Qu. Median Mean 3rd Qu. Max.
## 109.0 279.5 380.0 388.0 500.0 775.0

**summary**(rkts17)

## Min. 1st Qu. Median Mean 3rd Qu. Max.
## 95.0 312.8 349.0 337.2 376.0 424.0

**summary**(gts)

## Min. 1st Qu. Median Mean 3rd Qu. Max.
## 5.00 9.00 11.00 12.71 14.00 100.00

**summary**(gts17)

## Min. 1st Qu. Median Mean 3rd Qu. Max.
## 6.00 8.00 10.00 10.67 13.00 20.00

**library**(ggplot2)

p<-**ggplot**(data = masterall, **aes**(x = time)) **+**
 **geom_point**(**aes**(y = rkip, colour = "Weekly case numbers"), shape=0) **+**
 **stat_smooth**(**aes**(y = rkip, colour = "Weekly case numbers"), span=0.2, linetype="dashed")
 p<-p**+** **geom_point**(**aes**(y = pertugt*****10, colour = "Google search volume")) **+**
 **stat_smooth**(**aes**(y = pertugt*****10, colour = "Google search volume"), span=0.1) **+**
*# + scale_colour_manual("",*
 *# breaks = c("RKI", "GTD"),*
 *# values = c("red", "blue")) +*
 **scale_x_date**(date_minor_breaks = "1 month") **+**
 **xlab**(NULL) **+**
 **theme_bw**()
p<-p**+** **scale_y_continuous**(name= "Weekly case numbers", sec.axis= **sec_axis**( trans=**~**.**/**10, name= "Google search volume"))
p

## `geom_smooth()` using method = 'loess' and formula 'y ~ x'

## `geom_smooth()` using method = 'loess' and formula 'y ~ x'


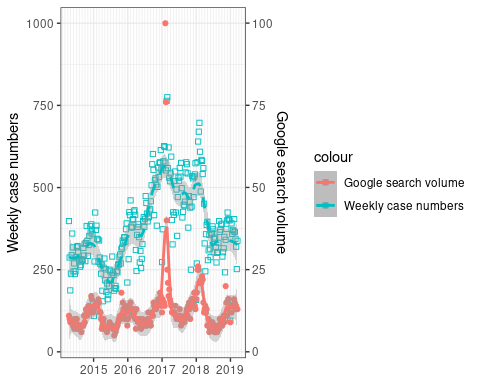


**par**(mfrow=**c**(2,2))

**acf**( rkts, lag.max = 52 )
**acf**( **diff**( rkts ), lag.max = 100 )
**pacf**( rkts, lag.max = 52 )
**pacf**( **diff**( rkts ), lag.max = 100 )


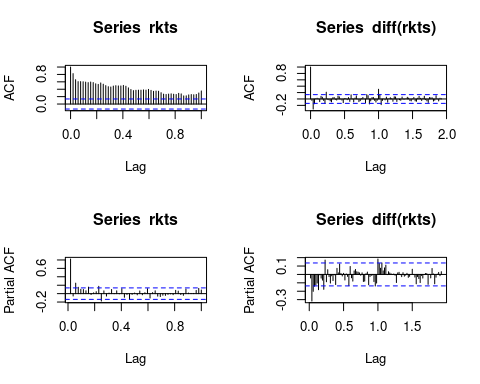


**par**(mfrow=**c**(2,2))
**acf**( **diff**( rkts, 52 ), lag.max = 100 )
**acf**( **diff**( **diff**( rkts, 52 ) ), lag.max = 100 )
**pacf**( **diff**( rkts, 52 ), lag.max = 100 )
**pacf**( **diff**( **diff**( rkts, 52 ) ), lag.max = 100 )


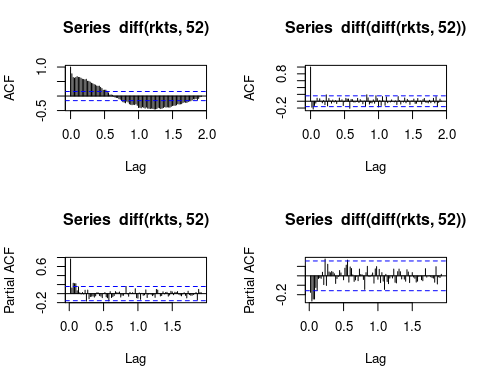


**library**(ggpubr)

##
## Attaching package: 'ggpubr'

## The following object is masked from 'package:forecast':
##
## gghistogram

**nsdiffs**(rkts)

## [1] 1

**nsdiffs**(**residuals**(rkts**~bs**( gts, knots = **c**( 9, 14 ), Boundary.knots = **c**( 0, 100 ) ) ))

## [1] 0

*#GTD with splines*
xreg<-**bs**( gts, knots = **quantile**(gts,(1**:**2)**/**3), Boundary.knots = **c**( 0, 100 ) )
nxreg<-**bs**( gts17, knots = **quantile**(gts,(1**:**2)**/**3), Boundary.knots = **c**( 0, 100 ) )


fit <- **auto.arima**( rkts, trace = TRUE, approximation = FALSE )

##
## ARIMA(2,1,2)(1,1,1)[52] : Inf
## ARIMA(0,1,0)(0,1,0)[52] : 1797.486
## ARIMA(1,1,0)(1,1,0)[52] : 1786.77
## ARIMA(0,1,1)(0,1,1)[52] : 1767.162
## ARIMA(0,1,1)(0,1,0)[52] : 1775.288
## ARIMA(0,1,1)(1,1,1)[52] : Inf
## ARIMA(0,1,1)(1,1,0)[52] : 1771.205
## ARIMA(0,1,0)(0,1,1)[52] : 1790.127
## ARIMA(1,1,1)(0,1,1)[52] : 1756.631
## ARIMA(1,1,1)(0,1,0)[52] : 1765.106
## ARIMA(1,1,1)(1,1,1)[52] : Inf
## ARIMA(1,1,1)(1,1,0)[52] : 1760.378
## ARIMA(1,1,0)(0,1,1)[52] : 1786.805
## ARIMA(2,1,1)(0,1,1)[52] : 1751.439
## ARIMA(2,1,1)(0,1,0)[52] : 1759.162
## ARIMA(2,1,1)(1,1,1)[52] : Inf
## ARIMA(2,1,1)(1,1,0)[52] : 1754.439
## ARIMA(2,1,0)(0,1,1)[52] : 1774.266
## ARIMA(3,1,1)(0,1,1)[52] : 1752.29
## ARIMA(2,1,2)(0,1,1)[52] : 1750.993
## ARIMA(2,1,2)(0,1,0)[52] : 1755.912
## ARIMA(2,1,2)(1,1,0)[52] : 1753.178
## ARIMA(1,1,2)(0,1,1)[52] : 1754.224
## ARIMA(3,1,2)(0,1,1)[52] : 1752.901
## ARIMA(2,1,3)(0,1,1)[52] : 1752.912
## ARIMA(1,1,3)(0,1,1)[52] : 1750.981
## ARIMA(1,1,3)(0,1,0)[52] : 1756.977
## ARIMA(1,1,3)(1,1,1)[52] : Inf
## ARIMA(1,1,3)(1,1,0)[52] : 1759.626
## ARIMA(0,1,3)(0,1,1)[52] : 1754.208
## ARIMA(1,1,4)(0,1,1)[52] : 1753.519
## ARIMA(0,1,2)(0,1,1)[52] : 1752.103
## ARIMA(0,1,4)(0,1,1)[52] : 1753.658
## ARIMA(2,1,4)(0,1,1)[52] : 1755.139
##
## Best model: ARIMA(1,1,3)(0,1,1)[52]

fit

## Series: rkts
## ARIMA(1,1,3)(0,1,1)[52]
##
## Coefficients:
## ar1 ma1 ma2 ma3 sma1
## 0.9490 -1.4497 0.0948 0.3783 -0.3320
## s.e. 0.0487 0.0847 0.1361 0.0745 0.1212
##
## sigma^2 estimated as 4601: log likelihood=-869.2
## AIC=1750.41 AICc=1750.98 BIC=1768.63

**summary**(fit)

## Series: rkts
## ARIMA(1,1,3)(0,1,1)[52]
##
## Coefficients:
## ar1 ma1 ma2 ma3 sma1
## 0.9490 -1.4497 0.0948 0.3783 -0.3320
## s.e. 0.0487 0.0847 0.1361 0.0745 0.1212
##
## sigma^2 estimated as 4601: log likelihood=-869.2
## AIC=1750.41 AICc=1750.98 BIC=1768.63
##
## Training set error measures:
## ME RMSE MAE MPE MAPE MASE
## Training set 2.707862 57.54623 36.39037 -0.4965793 9.642941 0.3260408
## ACF1
## Training set 0.03996512

**library**(lmtest)

## Loading required package: zoo

##
## Attaching package: 'zoo'

## The following objects are masked from 'package:base':
##
## as.Date, as.Date.numeric

**coeftest**(fit)

##
## z test of coefficients:
##
## Estimate Std. Error z value Pr(>|z|)
## ar1 0.949039 0.048688 19.4922 < 2.2e-16 ***
## ma1 -1.449665 0.084749 -17.1055 < 2.2e-16 ***
## ma2 0.094766 0.136110 0.6962 0.486275
## ma3 0.378284 0.074531 5.0755 3.864e-07 ***
## sma1 -0.332023 0.121220 -2.7390 0.006163 **
## ---
## Signif. codes: 0 '***' 0.001 '**' 0.01 '*' 0.05 '.' 0.1 ' ' 1

fc <- **forecast**( fit, h = **length**( gts17 ) )


fitGT <- **Arima**( rkts, order=**c**(1,1,3), seasonal=**c**(0,1,1), xreg = xreg )

fitGT

## Series: rkts
## Regression with ARIMA(1,1,3)(0,1,1)[52] errors
##
## Coefficients:
## ar1 ma1 ma2 ma3 sma1 1 2 3
## 0.6102 -1.1788 -0.0507 0.3500 -0.321 295.8108 208.8232 609.1758
## s.e. 0.1514 0.1417 0.1491 0.0776 0.120 546.0590 477.7267 493.1885
## 4 5
## 243.9820 259.7096
## s.e. 484.0184 481.8379
##
## sigma^2 estimated as 4326: log likelihood=-861.44
## AIC=1744.87 AICc=1746.73 BIC=1778.28

**summary**(fitGT)

## Series: rkts
## Regression with ARIMA(1,1,3)(0,1,1)[52] errors
##
## Coefficients:
## ar1 ma1 ma2 ma3 sma1 1 2 3
## 0.6102 -1.1788 -0.0507 0.3500 -0.321 295.8108 208.8232 609.1758
## s.e. 0.1514 0.1417 0.1491 0.0776 0.120 546.0590 477.7267 493.1885
## 4 5
## 243.9820 259.7096
## s.e. 484.0184 481.8379
##
## sigma^2 estimated as 4326: log likelihood=-861.44
## AIC=1744.87 AICc=1746.73 BIC=1778.28
##
## Training set error measures:
## ME RMSE MAE MPE MAPE MASE ACF1
## Training set 2.540483 54.85822 35.6462 -0.3800244 9.656539 0.3193735 0.04501622

**coeftest**(fitGT)

##
## z test of coefficients:
##
## Estimate Std. Error z value Pr(>|z|)
## ar1 0.610191 0.151449 4.0290 5.601e-05 ***
## ma1 -1.178767 0.141690 -8.3193 < 2.2e-16 ***
## ma2 -0.050730 0.149089 -0.3403 0.73365
## ma3 0.350048 0.077569 4.5127 6.401e-06 ***
## sma1 -0.320990 0.119950 -2.6760 0.00745 **
## 1 295.810788 546.059041 0.5417 0.58801
## 2 208.823188 477.726671 0.4371 0.66203
## 3 609.175776 493.188467 1.2352 0.21676
## 4 243.981986 484.018434 0.5041 0.61421
## 5 259.709641 481.837891 0.5390 0.58989
## ---
## Signif. codes: 0 '***' 0.001 '**' 0.01 '*' 0.05 '.' 0.1 ' ' 1

fcGT<- **forecast**( fitGT, xreg = nxreg )

*##plot- the models*

without<-**autoplot**(fc) **+** **autolayer**(rkts17) **+** **xlab**(NULL) **+** **ylab**("Reported daily pertussis cases") **+** **ggtitle**("Forecast of model without GTD") **+** **theme_bw**() **+** **theme**(legend.position = "none")

with<-**autoplot**(fcGT) **+** **autolayer**(rkts17) **+** **xlab**(NULL) **+** **ylab**("Reported daily pertussis cases") **+** **ggtitle**("Forecast of model with GTD") **+** **theme_bw**() **+** **theme**(legend.position = "none")

both<-**autoplot**(fc, PI=FALSE) **+** **autolayer**(rkts17) **+** **autolayer**(fcGT, PI=FALSE, col="black") **+** **xlab**(NULL) **+** **ylab**("Reported daily pertussis cases") **+** **ggtitle**("Forecasts of both models") **+** **theme_bw**() **+** **theme**(legend.position = "none")

fig3<-**ggarrange**(with, without, both, ncol=1)
fig3


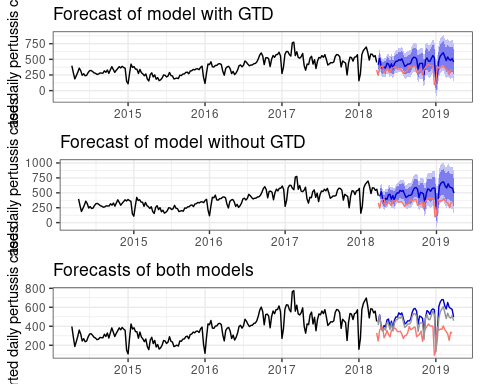


**layout**(1)
**plot**( fc, col = "black", ylab = "Number of cases", main = "")
**lines**( fcGT**$**mean, col = "red")
**lines**( rkts17, col = "black")
**legend**( "topleft", legend = **c**( "Optimal SARIMA", "Optimal SARIMA with Google Trends", "Actual" ),
 fill = **c**( "blue", "red", "black" ), cex = 0.7 )


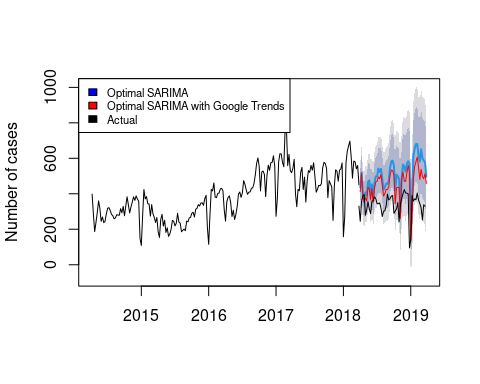


*# result:*
**accuracy**( fc, rkts17 )

## ME RMSE MAE MPE MAPE MASE
## Training set 2.707862 57.54623 36.39037 -0.4965793 9.642941 0.3260408
## Test set -165.429982 192.64697 169.52892 -57.5350532 58.591632 1.5189007
## ACF1 Theil's U
## Training set 0.03996512 NA
## Test set 0.38387981 1.793703

**accuracy**( fcGT, rkts17 )

## ME RMSE MAE MPE MAPE MASE
## Training set 2.540483 54.85822 35.6462 -0.3800244 9.656539 0.3193735
## Test set -114.028468 144.22093 124.4659 -40.9547180 43.858730 1.1151567
## ACF1 Theil's U
## Training set 0.04501622 NA
## Test set 0.19929541 1.346797

*##residuals*
**checkresiduals**(fit)


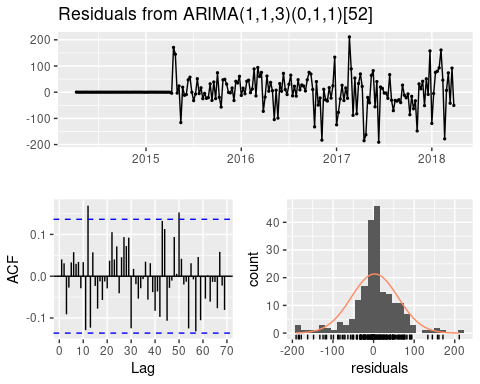


##
## Ljung-Box test
##
## data: Residuals from ARIMA(1,1,3)(0,1,1)[52]
## Q* = 40.469, df = 36, p-value = 0.2795
##
## Model df: 5. Total lags used: 41

**checkresiduals**(fitGT)


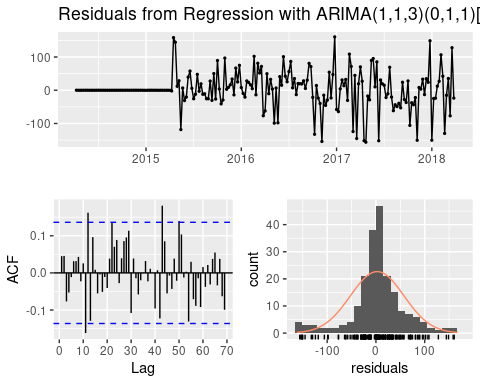


##
## Ljung-Box test
##
## data: Residuals from Regression with ARIMA(1,1,3)(0,1,1)[52] errors
## Q* = 45.188, df = 31, p-value = 0.04796
##
## Model df: 10. Total lags used: 41

fitGT

## Series: rkts
## Regression with ARIMA(1,1,3)(0,1,1)[52] errors
##
## Coefficients:
## ar1 ma1 ma2 ma3 sma1 1 2 3
## 0.6102 -1.1788 -0.0507 0.3500 -0.321 295.8108 208.8232 609.1758
## s.e. 0.1514 0.1417 0.1491 0.0776 0.120 546.0590 477.7267 493.1885
## 4 5
## 243.9820 259.7096
## s.e. 484.0184 481.8379
##
## sigma^2 estimated as 4326: log likelihood=-861.44
## AIC=1744.87 AICc=1746.73 BIC=1778.28

a<-**AIC**( fit, fitGT )
a

## df AIC
## fit 6 1750.409
## fitGT 11 1744.871

**dm.test**( rkts17**-**fc**$**mean, rkts17**-**fcGT**$**mean )

##
## Diebold-Mariano Test
##
## data: rkts17 - fc$meanrkts17 - fcGT$mean
## DM = 6.8627, Forecast horizon = 1, Loss function power = 2, p-value =
## 9.842e-09
## alternative hypothesis: two.sided

*##################################short term forecast#############################################*
gts <- **ts**( masterall**$**pertugt, start=**c**(2014, 15), end=**c**(2019, 12), frequency=52)
rkts <- **ts**( masterall**$**rkip , start=**c**(2014, 15), end=**c**(2019, 12), frequency=52)

gts17<-**ts**(gts[208**:**209], start=**c**(2018, 14), end=**c**(2018, 15), frequency=52)
rkts17<-**ts**(rkts[208**:**209], start=**c**(2018, 14), end=**c**(2018, 15), frequency=52)
nxreg<-**bs**( gts17, knots = **quantile**(gts,(1**:**2)**/**3), Boundary.knots = **c**( 0, 100 ) )

fcshort <- **forecast**( fit, h = **length**( gts17 ) )
fcGTshort<- **forecast**( fitGT, xreg = nxreg )


*# result:*
**accuracy**( fcshort, rkts17 )

## ME RMSE MAE MPE MAPE MASE
## Training set 2.707862 57.54623 36.39037 -0.4965793 9.642941 0.3260408
## Test set -190.532792 207.80682 190.53279 -72.1133100 72.113310 1.7070857
## ACF1 Theil's U
## Training set 0.03996512 NA
## Test set -0.50000000 3.217461

**accuracy**( fcGTshort, rkts17 )

## ME RMSE MAE MPE MAPE MASE
## Training set 2.540483 54.85822 35.6462 -0.3800244 9.656539 0.3193735
## Test set -178.963775 201.78352 178.9638 -68.5387923 68.538792 1.6034327
## ACF1 Theil's U
## Training set 0.04501622 NA
## Test set -0.50000000 3.202073

**dm.test**( rkts17**-**fcshort**$**mean, rkts17**-**fcGTshort**$**mean )

##
## Diebold-Mariano Test
##
## data: rkts17 - fcshort$meanrkts17 - fcGTshort$mean
## DM = 1.407, Forecast horizon = 1, Loss function power = 2, p-value =
## 0.3934
## alternative hypothesis: two.sided

*####evaluation on a rolling forecast origin##################*

gts <- **ts**( masterall**$**pertugt, start=**c**(2014, 15), end=**c**(2019, 12), frequency=52)
xreg<-**bs**( gts, knots = **quantile**(gts,(1**:**2)**/**3), Boundary.knots = **c**( 0, 100 ) )


rkts <- **ts**( masterall**$**rkip , start=**c**(2014, 15), end=**c**(2019, 12), frequency=52)


far2c <- **function**(x,h) {**forecast**(**Arima**(x, order=**c**(1,1,3), seasonal=**c**(0,1,1)), method = "ML", h=h)}
e<-**tsCV**(rkts, far2c, h=1, window= 100)
rmse<-**sqrt**(**mean**(e**^**2, na.rm=TRUE))
rmse

## [1] 84.11659

**library**("remotes")
remotes**::install_github**("robjhyndman/forecast")

## Downloading GitHub repo robjhyndman/forecast@HEAD

## curl (4.3 -> 4.3.2 ) [CRAN]
## zoo (1.8-8 -> 1.8-9 ) [CRAN]
## utf8 (1.1.4 -> 1.2.2 ) [CRAN]
## cli (2.5.0 -> 3.0.1 ) [CRAN]
## rlang (0.4.10 -> 0.4.11 ) [CRAN]
## pillar (1.6.1 -> 1.6.2 ) [CRAN]
## fansi (0.4.1 -> 0.5.0 ) [CRAN]
## colorspace (2.0-0 -> 2.0-2 ) [CRAN]
## viridisLite (0.3.0 -> 0.4.0 ) [CRAN]
## farver (2.0.3 -> 2.1.0 ) [CRAN]
## Rcpp (1.0.6 -> 1.0.7 ) [CRAN]
## withr (2.3.0 -> 2.4.2 ) [CRAN]
## tibble (3.1.2 -> 3.1.3 ) [CRAN]
## isoband (0.2.3 -> 0.2.5 ) [CRAN]
## RcppArmad... (0.10.1.2.2 -> 0.10.6.0.0) [CRAN]
## ggplot2 (3.3.3 -> 3.3.5 ) [CRAN]

## Installing 16 packages: curl, zoo, utf8, cli, rlang, pillar, fansi, colorspace, viridisLite, farver, Rcpp, withr, tibble, isoband, RcppArmadillo, ggplot2

## Installing packages into '/home/sulyokm/R/x86_64-pc-linux-gnu-library/4.0'
## (as 'lib' is unspecified)

## checking for file ‘/tmp/RtmpU2Zlvz/remotes23c42243cd05/robjhyndman-forecast-d7ce2fb/DESCRIPTION’ ... ✓ checking for file ‘/tmp/RtmpU2Zlvz/remotes23c42243cd05/robjhyndman-forecast-d7ce2fb/DESCRIPTION’ (393ms)
## ─ preparing ‘forecast’:
## checking DESCRIPTION meta-information ... ✓ checking DESCRIPTION meta-information
## ─ cleaning src
## ─ checking for LF line-endings in source and make files and shell scripts
## ─ checking for empty or unneeded directories
## ─ building ‘forecast_8.15.0.9000.tar.gz’
##
##

## Installing package into '/home/sulyokm/R/x86_64-pc-linux-gnu-library/4.0'
## (as 'lib' is unspecified)

far2e <- **function**(x,h, xreg, newxreg) {**forecast**(**Arima**(x, order=**c**(1,1,3),seasonal=**c**(0,1,1), xreg = xreg, method = "ML"), h=h, xreg = newxreg)}
e2<-**tsCV**(rkts, far2e, h=1, window= 100, xreg = xreg)

rmsegt<-**sqrt**(**mean**(e2**^**2, na.rm=TRUE))
rmsegt

## [1] 30490.69

**autoplot**(e, series="SARIMA with traditional data", alpha=0.6) **+** **geom_smooth**(col="blue") **+**
 **autolayer**(e2, series="GTD expanded SARIMA", alpha=0.6) **+** **stat_smooth**(**aes**(y=e2), col="red") **+** **ylab**("Errors") **+** **theme_bw**() **+** **geom_hline**(yintercept = 0)

## `geom_smooth()` using method = 'loess' and formula 'y ~ x'

## Warning: Removed 109 rows containing non-finite values (stat_smooth).

## `geom_smooth()` using method = 'loess' and formula 'y ~ x'

## Warning: Removed 102 rows containing non-finite values (stat_smooth).

## Warning: Removed 100 row(s) containing missing values (geom_path).


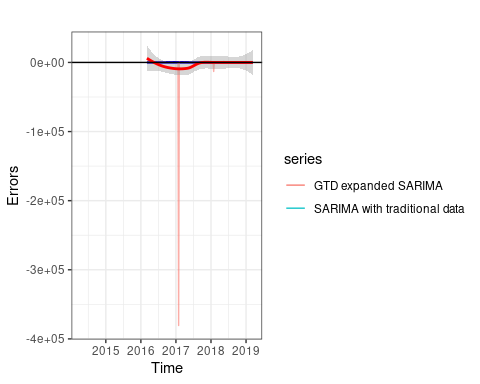


*#############checking Arima( rkts, order=c(2,0,2), seasonal=c(1,0,0))####################*

*##2 0 2 1 0 0 ARIMA from zhang et al*


masterall <- **read_delim**("pertussismeaslesmaster.csv", ";", escape_double = FALSE, col_types = **cols**(time = **col_date**(format = "%Y.%m.%d")), trim_ws = TRUE)


*#masterall[is.na(masterall$rkip)] <- 0*
**summary**(masterall)

## time pertugt impgt mgt
## Min. :2014-04-13 Min. : 5.0 Min. : 7.00 Min. : 1.000
## 1st Qu.:2015-07-06 1st Qu.: 9.0 1st Qu.:15.00 1st Qu.: 2.000
## Median :2016-09-28 Median : 11.0 Median :16.00 Median : 2.000
## Mean :2016-09-28 Mean : 12.3 Mean :16.37 Mean : 3.341
## 3rd Qu.:2017-12-22 3rd Qu.: 14.0 3rd Qu.:18.00 3rd Qu.: 3.000
## Max. :2019-03-17 Max. :100.0 Max. :42.00 Max. :100.000
## rkip
## Min. : 95.0
## 1st Qu.:284.8
## Median :365.0
## Mean :378.0
## 3rd Qu.:450.2
## Max. :775.0

**cor.test**(masterall**$**rkip, masterall**$**pertugt, method="kendall")

##
## Kendall's rank correlation tau
##
## data: masterall$rkip and masterall$pertugt
## z = 9.1644, p-value < 2.2e-16
## alternative hypothesis: true tau is not equal to 0
## sample estimates:
## tau
## 0.3980472

gts <- **ts**( masterall**$**pertugt, start=**c**(2014, 15), end=**c**(2019, 12), frequency=52)


rkts <- **ts**( masterall**$**rkip , start=**c**(2014, 15), end=**c**(2019, 12), frequency=52)

gts17<-**ts**(gts[208**:**258], start=**c**(2018, 13), end=**c**(2019, 12), frequency=52)
gts<-**ts**(gts[54**:**207], start=**c**(2015, 16), end=**c**(2018, 13), frequency=52)

rkts17<-**ts**(rkts[208**:**258], start=**c**(2018, 13), end=**c**(2019, 12), frequency=52)
rkts<-**ts**(rkts[54**:**207], start=**c**(2015, 16), end=**c**(2018, 13), frequency=52)

*#GTD with splines*
xreg<-**bs**( gts, knots = **quantile**(gts,(1**:**2)**/**3), Boundary.knots = **c**( 0, 100 ) )
nxreg<-**bs**( gts17, knots = **quantile**(gts,(1**:**2)**/**3), Boundary.knots = **c**( 0, 100 ) )


fitz <- **Arima**( rkts, order=**c**(2,0,2), seasonal=**c**(1,0,0))
fitz

## Series: rkts
## ARIMA(2,0,2)(1,0,0)[52] with non-zero mean
##
## Coefficients:
## ar1 ar2 ma1 ma2 sar1 mean
## 1.1461 -0.1566 -0.4969 -0.3014 0.4723 368.9735
## s.e. 0.1740 0.1710 0.1633 0.1198 0.0822 126.2977
##
## sigma^2 estimated as 4733: log likelihood=-874.75
## AIC=1763.5 AICc=1764.27 BIC=1784.76

**summary**(fitz)

## Series: rkts
## ARIMA(2,0,2)(1,0,0)[52] with non-zero mean
##
## Coefficients:
## ar1 ar2 ma1 ma2 sar1 mean
## 1.1461 -0.1566 -0.4969 -0.3014 0.4723 368.9735
## s.e. 0.1740 0.1710 0.1633 0.1198 0.0822 126.2977
##
## sigma^2 estimated as 4733: log likelihood=-874.75
## AIC=1763.5 AICc=1764.27 BIC=1784.76
##
## Training set error measures:
## ME RMSE MAE MPE MAPE MASE
## Training set 6.066011 67.44215 50.0067 -1.864914 13.80306 0.3676698
## ACF1
## Training set -0.004387361

**coeftest**(fitz)

##
## z test of coefficients:
##
## Estimate Std. Error z value Pr(>|z|)
## ar1 1.146147 0.174046 6.5853 4.539e-11 ***
## ar2 -0.156625 0.171003 -0.9159 0.359710
## ma1 -0.496853 0.163281 -3.0429 0.002343 **
## ma2 -0.301409 0.119831 -2.5153 0.011894 *
## sar1 0.472289 0.082205 5.7452 9.180e-09 ***
## intercept 368.973499 126.297728 2.9215 0.003484 **
## ---
## Signif. codes: 0 '***' 0.001 '**' 0.01 '*' 0.05 '.' 0.1 ' ' 1

fcz <- **forecast**( fitz, h = **length**( gts17 ) )


fitGTz <- **Arima**( rkts, order=**c**(2,0,2), seasonal=**c**(1,0,0), xreg = xreg)

fitGTz

## Series: rkts
## Regression with ARIMA(2,0,2)(1,0,0)[52] errors
##
## Coefficients:
## ar1 ar2 ma1 ma2 sar1 intercept 1 2
## 0.9910 -0.0019 -0.4516 -0.3442 0.4957 70.4411 241.2216 217.6141
## s.e. 0.0374 0.0346 0.1117 0.0964 0.0613 569.2157 627.0399 552.0589
## 3 4 5
## 646.9916 189.8905 257.0336
## s.e. 578.0314 562.4617 558.4447
##
## sigma^2 estimated as 4310: log likelihood=-865.69
## AIC=1755.37 AICc=1757.59 BIC=1791.82

**summary**(fitGTz)

## Series: rkts
## Regression with ARIMA(2,0,2)(1,0,0)[52] errors
##
## Coefficients:
## ar1 ar2 ma1 ma2 sar1 intercept 1 2
## 0.9910 -0.0019 -0.4516 -0.3442 0.4957 70.4411 241.2216 217.6141
## s.e. 0.0374 0.0346 0.1117 0.0964 0.0613 569.2157 627.0399 552.0589
## 3 4 5
## 646.9916 189.8905 257.0336
## s.e. 578.0314 562.4617 558.4447
##
## sigma^2 estimated as 4310: log likelihood=-865.69
## AIC=1755.37 AICc=1757.59 BIC=1791.82
##
## Training set error measures:
## ME RMSE MAE MPE MAPE MASE
## Training set 5.398093 63.26048 47.90958 -1.864016 13.42275 0.3522509
## ACF1
## Training set -0.007900666

**coeftest**(fitGTz)

##
## z test of coefficients:
##
## Estimate Std. Error z value Pr(>|z|)
## ar1 0.9910221 0.0374142 26.4878 < 2.2e-16 ***
## ar2 -0.0019265 0.0345939 -0.0557 0.9555896
## ma1 -0.4515598 0.1117466 -4.0409 5.324e-05 ***
## ma2 -0.3441921 0.0963511 -3.5723 0.0003539 ***
## sar1 0.4957268 0.0612913 8.0880 6.063e-16 ***
## intercept 70.4411232 569.2157437 0.1238 0.9015123
## 1 241.2216120 627.0399147 0.3847 0.7004605
## 2 217.6140601 552.0588941 0.3942 0.6934435
## 3 646.9916491 578.0313552 1.1193 0.2630113
## 4 189.8905409 562.4617329 0.3376 0.7356600
## 5 257.0335881 558.4447204 0.4603 0.6453247
## ---
## Signif. codes: 0 '***' 0.001 '**' 0.01 '*' 0.05 '.' 0.1 ' ' 1

fcGTz<- **forecast**( fitGTz, xreg = nxreg )

*# result:*

**accuracy**( fcz, rkts17 )

## ME RMSE MAE MPE MAPE MASE
## Training set 6.066011 67.44215 50.0067 -1.864914 13.80306 0.3676698
## Test set -129.478620 146.62582 130.9141 -47.024803 47.39194 0.9625345
## ACF1 Theil's U
## Training set -0.004387361 NA
## Test set 0.147922746 1.526193

**accuracy**( fcGTz, rkts17 )

## ME RMSE MAE MPE MAPE MASE
## Training set 5.398093 63.26048 47.90958 -1.864016 13.42275 0.3522509
## Test set -84.020023 108.69353 89.86374 -32.370928 33.93862 0.6607152
## ACF1 Theil's U
## Training set -0.007900666 NA
## Test set 0.115448628 1.05328

*##residuals*
**checkresiduals**(fitz)


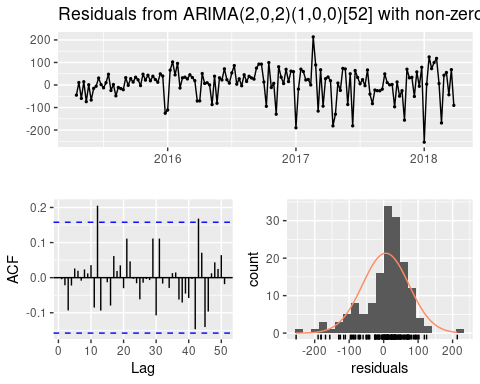


##
## Ljung-Box test
##
## data: Residuals from ARIMA(2,0,2)(1,0,0)[52] with non-zero mean
## Q* = 24.599, df = 25, p-value = 0.485
##
## Model df: 6. Total lags used: 31

**checkresiduals**(fitGTz)


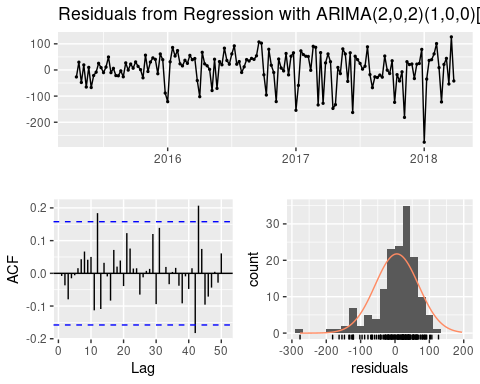


##
## Ljung-Box test
##
## data: Residuals from Regression with ARIMA(2,0,2)(1,0,0)[52] errors
## Q* = 28.88, df = 20, p-value = 0.09015
##
## Model df: 11. Total lags used: 31

fitGT

## Series: rkts
## Regression with ARIMA(1,1,3)(0,1,1)[52] errors
##
## Coefficients:
## ar1 ma1 ma2 ma3 sma1 1 2 3
## 0.6102 -1.1788 -0.0507 0.3500 -0.321 295.8108 208.8232 609.1758
## s.e. 0.1514 0.1417 0.1491 0.0776 0.120 546.0590 477.7267 493.1885
## 4 5
## 243.9820 259.7096
## s.e. 484.0184 481.8379
##
## sigma^2 estimated as 4326: log likelihood=-861.44
## AIC=1744.87 AICc=1746.73 BIC=1778.28

a<-**AIC**( fit, fitGT, fitz, fitGTz )
a

## df AIC
## fit 6 1750.409
## fitGT 11 1744.871
## fitz 7 1763.502
## fitGTz 12 1755.374

**dm.test**( rkts17**-**fc**$**mean, rkts17**-**fcz**$**mean )

##
## Diebold-Mariano Test
##
## data: rkts17 - fc$meanrkts17 - fcz$mean
## DM = 4.7574, Forecast horizon = 1, Loss function power = 2, p-value =
## 1.708e-05
## alternative hypothesis: two.sided

**dm.test**( rkts17**-**fcGT**$**mean, rkts17**-**fcGTz**$**mean )

##
## Diebold-Mariano Test
##
## data: rkts17 - fcGT$meanrkts17 - fcGTz$mean
## DM = 6.0652, Forecast horizon = 1, Loss function power = 2, p-value =
## 1.733e-07
## alternative hypothesis: two.sided

*#############checking Arima( rkts, order=c(1,0,2), seasonal=c(1,0,0), xreg = xreg )####################*

*##1 0 2 1 0 0 ARIMA*


fitz <- **Arima**( rkts, order=**c**(1,0,2), seasonal=**c**(1,0,0))
fitz

## Series: rkts
## ARIMA(1,0,2)(1,0,0)[52] with non-zero mean
##
## Coefficients:

## Warning in sqrt(diag(x$var.coef)): NaNs produced

## ar1 ma1 ma2 sar1 mean
## 0.9955 -0.3784 -0.3891 0.4799 584.6325
## s.e. NaN 0.0716 0.0690 0.0818 NaN
##
## sigma^2 estimated as 4724: log likelihood=-876.05
## AIC=1764.09 AICc=1764.66 BIC=1782.31

**summary**(fitz)

## Series: rkts
## ARIMA(1,0,2)(1,0,0)[52] with non-zero mean
##
## Coefficients:

## Warning in sqrt(diag(x$var.coef)): NaNs produced

## ar1 ma1 ma2 sar1 mean
## 0.9955 -0.3784 -0.3891 0.4799 584.6325
## s.e. NaN 0.0716 0.0690 0.0818 NaN
##
## sigma^2 estimated as 4724: log likelihood=-876.05
## AIC=1764.09 AICc=1764.66 BIC=1782.31
##
## Training set error measures:
## ME RMSE MAE MPE MAPE MASE ACF1
## Training set 1.83936 67.60821 49.41114 -2.836489 13.83549 0.363291 0.02661007

**coeftest**(fitz)

## Warning in sqrt(diag(se)): NaNs produced

##
## z test of coefficients:
##
## Estimate Std. Error z value Pr(>|z|)
## ar1 0.995548 NA NA NA
## ma1 -0.378417 0.071605 -5.2848 1.259e-07 ***
## ma2 -0.389080 0.069034 -5.6361 1.740e-08 ***
## sar1 0.479868 0.081752 5.8698 4.364e-09 ***
## intercept 584.632472 NA NA NA
## ---
## Signif. codes: 0 '***' 0.001 '**' 0.01 '*' 0.05 '.' 0.1 ' ' 1

fcz <- **forecast**( fitz, h = **length**( gts17 ) )


fitGTz <- **Arima**( rkts, order=**c**(1,0,2), seasonal=**c**(1,0,0), xreg = xreg )

fitGTz

## Series: rkts
## Regression with ARIMA(1,0,2)(1,0,0)[52] errors
##
## Coefficients:
## ar1 ma1 ma2 sar1 intercept 1 2 3
## 0.9878 -0.4504 -0.3416 0.4982 97.1061 227.2604 206.8578 639.3016
## s.e. 0.0109 0.0802 0.0746 0.0811 569.4938 628.2955 553.1723 579.1248
## 4 5
## 179.9610 246.6119
## s.e. 563.5893 559.7329
##
## sigma^2 estimated as 4278: log likelihood=-865.67
## AIC=1753.35 AICc=1755.21 BIC=1786.75

**summary**(fitGTz)

## Series: rkts
## Regression with ARIMA(1,0,2)(1,0,0)[52] errors
##
## Coefficients:
## ar1 ma1 ma2 sar1 intercept 1 2 3
## 0.9878 -0.4504 -0.3416 0.4982 97.1061 227.2604 206.8578 639.3016
## s.e. 0.0109 0.0802 0.0746 0.0811 569.4938 628.2955 553.1723 579.1248
## 4 5
## 179.9610 246.6119
## s.e. 563.5893 559.7329
##
## sigma^2 estimated as 4278: log likelihood=-865.67
## AIC=1753.35 AICc=1755.21 BIC=1786.75
##
## Training set error measures:
## ME RMSE MAE MPE MAPE MASE
## Training set 5.127907 63.24468 47.91334 -1.977156 13.43653 0.3522786
## ACF1
## Training set -0.007637669

**coeftest**(fitGTz)

##
## z test of coefficients:
##
## Estimate Std. Error z value Pr(>|z|)
## ar1 0.987840 0.010937 90.3240 < 2.2e-16 ***
## ma1 -0.450414 0.080240 -5.6133 1.985e-08 ***
## ma2 -0.341604 0.074645 -4.5764 4.731e-06 ***
## sar1 0.498184 0.081120 6.1413 8.186e-10 ***
## intercept 97.106111 569.493757 0.1705 0.8646
## 1 227.260414 628.295471 0.3617 0.7176
## 2 206.857813 553.172286 0.3739 0.7084
## 3 639.301640 579.124799 1.1039 0.2696
## 4 179.960958 563.589279 0.3193 0.7495
## 5 246.611928 559.732851 0.4406 0.6595
## ---
## Signif. codes: 0 '***' 0.001 '**' 0.01 '*' 0.05 '.' 0.1 ' ' 1

fcGTz<- **forecast**( fitGTz, xreg = nxreg )

*# result:*
**accuracy**( fc, rkts17 )

## ME RMSE MAE MPE MAPE MASE
## Training set 2.707862 57.54623 36.39037 -0.4965793 9.642941 0.3260408
## Test set -165.429982 192.64697 169.52892 -57.5350532 58.591632 1.5189007
## ACF1 Theil's U
## Training set 0.03996512 NA
## Test set 0.38387981 1.793703

**accuracy**( fcGT, rkts17 )

## ME RMSE MAE MPE MAPE MASE
## Training set 2.540483 54.85822 35.6462 -0.3800244 9.656539 0.3193735
## Test set -114.028468 144.22093 124.4659 -40.9547180 43.858730 1.1151567
## ACF1 Theil's U
## Training set 0.04501622 NA
## Test set 0.19929541 1.346797

**accuracy**( fcz, rkts17 )

## ME RMSE MAE MPE MAPE MASE
## Training set 1.83936 67.60821 49.41114 -2.836489 13.83549 0.363291
## Test set -155.18979 170.50831 155.27132 -55.297420 55.31827 1.141619
## ACF1 Theil's U
## Training set 0.02661007 NA
## Test set 0.18720795 1.79275

**accuracy**( fcGTz, rkts17 )

## ME RMSE MAE MPE MAPE MASE
## Training set 5.127907 63.24468 47.91334 -1.977156 13.43653 0.3522786
## Test set -85.165414 109.77025 90.89430 -32.738707 34.27608 0.6682922
## ACF1 Theil's U
## Training set -0.007637669 NA
## Test set 0.118916710 1.062207

*##residuals*
**checkresiduals**(fitz)


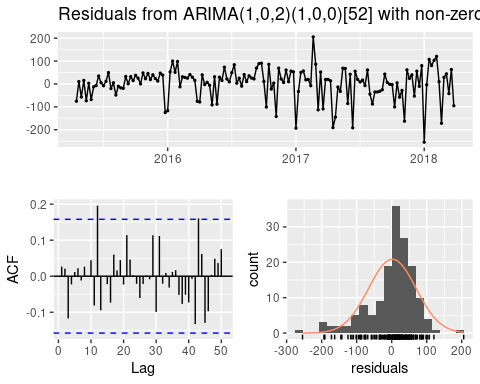


##
## Ljung-Box test
##
## data: Residuals from ARIMA(1,0,2)(1,0,0)[52] with non-zero mean
## Q* = 24.703, df = 26, p-value = 0.5358
##
## Model df: 5. Total lags used: 31

**checkresiduals**(fitGTz)


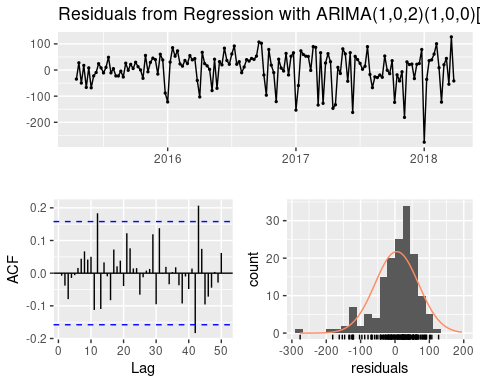


##
## Ljung-Box test
##
## data: Residuals from Regression with ARIMA(1,0,2)(1,0,0)[52] errors
## Q* = 28.776, df = 21, p-value = 0.1195
##
## Model df: 10. Total lags used: 31

fitGT

## Series: rkts
## Regression with ARIMA(1,1,3)(0,1,1)[52] errors
##
## Coefficients:
## ar1 ma1 ma2 ma3 sma1 1 2 3
## 0.6102 -1.1788 -0.0507 0.3500 -0.321 295.8108 208.8232 609.1758
## s.e. 0.1514 0.1417 0.1491 0.0776 0.120 546.0590 477.7267 493.1885
## 4 5
## 243.9820 259.7096
## s.e. 484.0184 481.8379
##
## sigma^2 estimated as 4326: log likelihood=-861.44
## AIC=1744.87 AICc=1746.73 BIC=1778.28

a<-**AIC**( fit, fitGT, fitz, fitGTz )
a

## df AIC
## fit 6 1750.409
## fitGT 11 1744.871
## fitz 6 1764.091
## fitGTz 11 1753.347

**dm.test**( rkts17**-**fc**$**mean, rkts17**-**fcz**$**mean )

##
## Diebold-Mariano Test
##
## data: rkts17 - fc$meanrkts17 - fcz$mean
## DM = 3.0172, Forecast horizon = 1, Loss function power = 2, p-value =
## 0.004005
## alternative hypothesis: two.sided

**dm.test**( rkts17**-**fcGT**$**mean, rkts17**-**fcGTz**$**mean )

##
## Diebold-Mariano Test
##
## data: rkts17 - fcGT$meanrkts17 - fcGTz$mean
## DM = 6.0539, Forecast horizon = 1, Loss function power = 2, p-value =
## 1.805e-07
## alternative hypothesis: two.sided

**dm.test**( rkts17**-**fcz**$**mean, rkts17**-**fcGTz**$**mean)

##
## Diebold-Mariano Test
##
## data: rkts17 - fcz$meanrkts17 - fcGTz$mean
## DM = 10.93, Forecast horizon = 1, Loss function power = 2, p-value =
## 7.416e-15
## alternative hypothesis: two.sided
